# Outcome Analysis of Speed Gate Cannulation during Standard Infrarenal Endovascular Aneurysm Repair

**DOI:** 10.3390/jcm12196263

**Published:** 2023-09-28

**Authors:** Domenico Mirabella, Salvatore Evola, Ettore Dinoto, Carlo Setacci, David Pakeliani, Francesco Setacci, Paolo Annicchiarico, Felice Pecoraro

**Affiliations:** 1 Vascular Surgery Unit, AOU Policlinico “P. Giaccone”, 90127 Palermo, Italy; dmirabella@live.it (D.M.); annicchiarico.paolo@gmail.com (P.A.); felice.pecoraro@unipa.it (F.P.); 2 Cardiology Unit, AOUP Policlinico “P. Giaccone”, 90127 Palermo, Italy; cardioevola@gmail.com; 3 Vascular Surgery Unit, University of Siena, 53100 Siena, Italy; carlo.setacci@unisi.it; 4 Vascular Surgery Unit, Ospedali Riuniti Villa Sofia-Cervello, 90146 Palermo, Italy; davidpakeliani@gmail.com; 5 Vascular Surgery Unit, Università degli Studi di Enna “Kore”, 94100 Enna, Italy; francesco.setacci@unikore.it; 6 IRCCS MultiMedica, 20138 Milan, Italy; 7 Department of Surgical Oncological and Oral Sciences, University of Palermo, 90128 Palermo, Italy

**Keywords:** speed gate cannulation, contralateral limb, gate, cannulation, EVAR, abdominal aortic aneurysm

## Abstract

Background: Endovascular aortic repair (EVAR) is generally performed with bi/trimodular stent-grafts requiring retrograde contralateral gate cannulation (CGC). In the case of tricky CGC, an increased EVAR procedural time and radiation exposure have been reported. Herein, we compare the outcomes of conventional CGC and CGC using the speed gate cannulation (SGC) technique in standard EVAR for a propensity-matched cohort. Methods: A total of 371 patients were retrospectively analyzed. Inclusion criteria were fulfilled in 172 patients who underwent propensity score matching. Primary outcomes included operative time, CGC time, mean contrast medium, fluoroscopy time, and CGC fluoroscopy time. Results: After matching, 78 patients were included in each group (SGC vs. standard). Primary outcomes registered a significant reduction in CGC time (4 [1–6] vs. 8 [6–14] min; *p* = 0.001) and fluoroscopy time (12 [9–16] vs. 17 [12–25] min). Conclusions: In this preliminary experiment, the use of SGC was feasible with no significant registered postoperative complications. A significant reduction in contrast medium usage, radiation exposure, and CGC time was observed with the use of SGC. SGC is a simple adjunctive technique, and its use should be considered in standard EVAR, especially in emergency scenarios, where time is of the essence.

## 1. Introduction

The endovascular aortic repair (EVAR) of abdominal aortic aneurysms (AAAs) with bi- or tri-modular stent-grafts involves multiple steps, including contralateral gate cannulation (CGC) [1]. Standard CGC is carried out in a retrograde fashion through contralateral femoral access. Different AAA anatomic variables have been associated with difficult standard CGC and increased EVAR procedural times, radiation exposure, and endovascular material costs for a bailout [2]. To facilitate CGC, we usually resort to the speed-gate cannulation technique (SGC), a tool that reduces the time of this step by allowing us to open the gate near to the guide wire coming from the contralateral access. Herein, we compare the outcomes of conventional CGC and CGC using the SGC technique in standard EVAR for a propensity-matched cohort.

## 2. Materials and Methods

From February 2017 to December 2022, patients treated with standard EVAR using bi- or tri-modular stent-grafts to address AAAs were retrospectively analyzed. Of the 371 patients analyzed, conventional CGC was used in 151 (40.7%) patients and SGC in 220 (59.3%) patients. Since 2015, SGC has been employed at our institution as a standard step for all patients undergoing EVAR for AAAs.

A total of 199 patients were excluded for being treated in a non-elective setting, with aortouniliac EVAR, EVAR relining, complex EVAR, or EVAR after a previous surgical intervention. The remaining 172 patients were considered eligible for inclusion (first procedure for AAA in an elective setting without the need for additional treatments beyond implantation of the aortic prosthesis); 83 (48.5%) patients were treated with a conventional CGC and 89 (51.5%) using SGC. Patients were matched using the propensity score method to obtain 2 homogeneous groups of patients treated using conventional CGC and SGC.

All patients included in the study gave informed consent for the procedure itself, anonymous data collection, and analysis. According to the Institutional Review Board, the retrospective and anonymized nature of the study did not require medical ethics committee approval. The study was performed in agreement with the Declaration of Helsinki and followed the STROBE guidelines for reporting observational studies [3].

Primary outcomes included operative time, CGC time, mean contrast medium, fluoroscopy time, dose-area product (DAP), and CGC fluoroscopy time. Secondary outcomes were perioperative mortality and morbidity, endoleak incidence, number of iliac stent-grafts, survival, and freedom from reintervention. Technical success was defined as endograft deployment in the intended position and no angiographic type I or III endoleaks, or limb occlusion within 24 h after the EVAR.

Before the procedure, every patient was studied with CTA. The same imaging was employed for follow-up, including a CTA performed at 2 and 12 months and duplex ultrasound at 6 months. In patients with renal function impairment, a standard computed tomography (CT) and contrast-enhanced ultrasound replaced CTA after the same time interval.

SGC Technique. Until contraindicated and according to the instruction for use, a bilateral percutaneous approach using a 6 Fr Prostyle vascular closure device (Abbott Vascular, Redwood City, CA, USA) was preferred. After preclosing, a 10 Fr introducer sheath was inserted over the access guidewire bilaterally. A 5 Fr Multipurpose catheter was introduced to facilitate guidewire exchange to a 0.035-inch stiff wire (Backup Meier or Amplatz, Boston Scientific, Natick, MA, USA).

**Figure 1:**
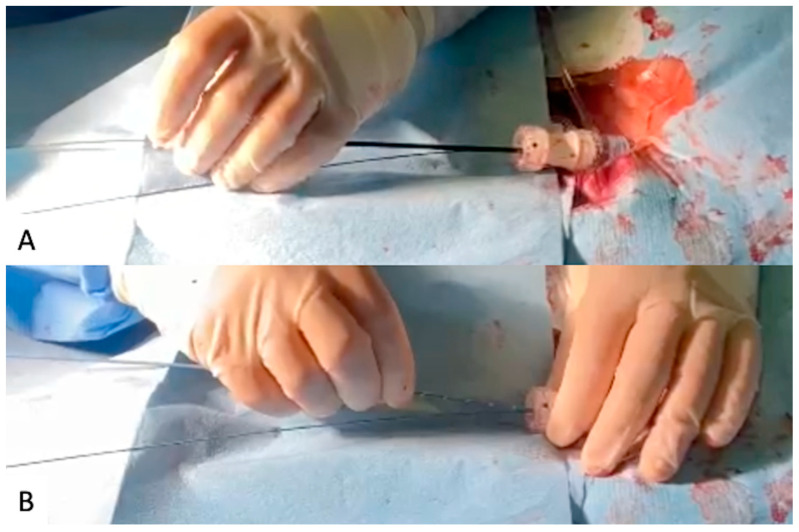
(**A**) Short dilator and parallel 0.035-inch standard J-tip guide-wire through the Dryseal introducer. (**B**) A 5 Fr pigtail angiographic catheter advancement.

The contralateral 10 Fr sheath on the intended main body introduction side was exchanged for a 30 cm 12–18 Fr Dryseal introducer sheath (W. L. Gore & Associates, Medical Products Division, Flagstaff, AZ, USA) over the stiff wire and advanced into the aorta at the level of the lowest renal artery. A short dilator was employed to place it parallel to the stiff wire; an additional 0.035-inch standard J-tip guidewire was applied through the Dryseal introducer. A 5 Fr pigtail angiographic catheter was advanced over the standard J-tip guidewire into the aorta above the level of the renal arteries (Figure 1).

**Figure 2:**
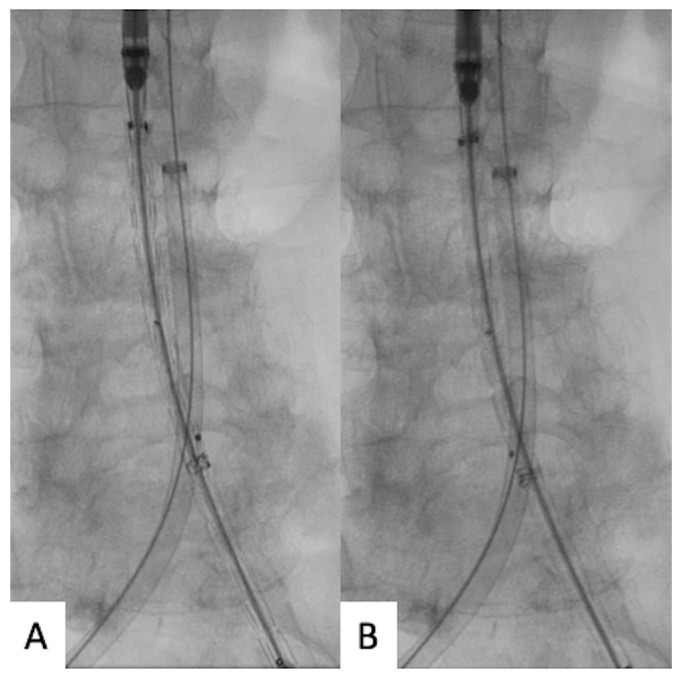
(**A**) Main body introduction before orientation towards the contralateral supported sheath route. (**B**) Main body rotation to orientate the distal markers of the contralateral gate in the direction of the route of the contralateral wires.

Through the main access, the main body was advanced and positioned at the level of the renal arteries. At this point, the positioned main body was rotated to orientate the distal markers of the contralateral gate in the direction of the route of the contralateral wires. The aim of the orientation is to allow the contralateral gate to open as close as possible to the contralateral wireless route (Figure 2).

**Figure 3:**
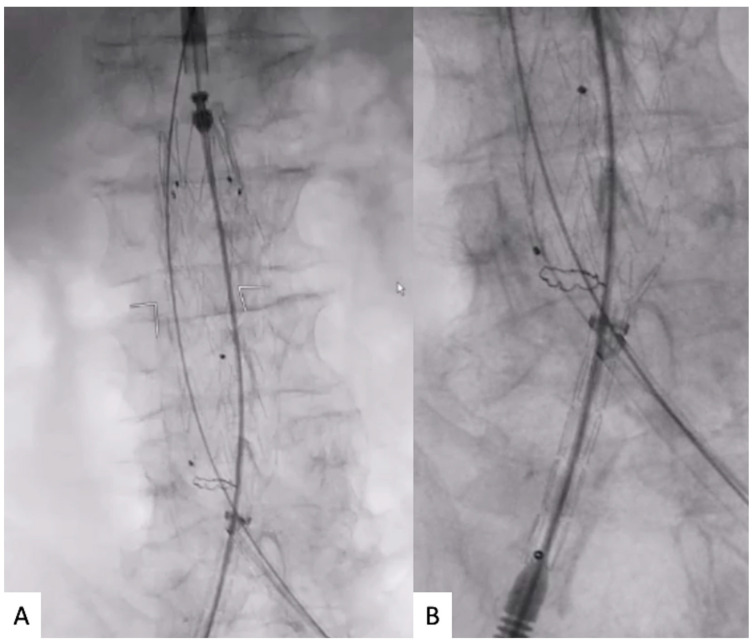
(**A**) Contralateral supported sheath lowering below the contralateral gate radiopaque markers and main body deployment in ballerina configuration. (**B**) Contralateral gate cannulation with buddy wire through the contralateral supported sheath.

Then, the Dryseal introducer was lowered just below the markers of the contralateral gate; an aortography was performed from the placed 5 Fr pigtail, to visualize the renal arteries; and the stent-graft main body was deployed in a crossed limb “ballerina” or standard configuration depending on the previous orientation. Such steps aim to obtain a supported sheath as close as possible to the contralateral gate opening, to speed up the CGC (Figure 3A). A standard J-tip guidewire is inserted into the 5 Fr pigtail to lower it just below the opening of the contralateral gate at the same level as the 16 Fr Dryseal introducer tip. The CGC begins using the same 5 Fr pigtail and the standard J-tip guidewire; both the J-tip guidewire and 5 Fr pigtail can be exchanged to facilitate CGC (Figure 3B).

After CGC, the 5 Fr pigtail was advanced over the J-tip guidewire up to the ascending aorta, the coaxial stiff wire was withdrawn and exchanged with the J-tip guidewire into the 5 Fr pigtail. Subsequent EVAR steps were as standard, according to the device instructions for use.

Statistical Analysis. Propensity score matching was performed to obtain 2 homogeneous groups in terms of age, sex, and associated comorbidities (hypertension, ever smoker, chronic obstructive pulmonary disease, cerebrovascular disease, peripheral arterial disease [PAD], diabetes mellitus [DM], coronary disease, lipid disorder), previous cardiac interventions, preoperative GFR < 60 mL/min, left ventricle ejection fraction < 50%, preoperative New York Heart Association (NYHA) classification, American Society of Anesthesiologists (ASA) classification, type of anesthesia, aneurysm behavior (maximal diameter, neck length, neck angle, neck diameter, common iliac artery (CIA) involvement, and aortic carrefour angulation on coronal axis), and operation details (stent-graft fabric, number of components, and “ballerina” configuration).

Categorical variables were reported as absolute number and frequency (%) and compared using the chi-squared or Fisher’s exact test. Continuous data were reported as the median [interquartile range] and compared using the Mann–Whitney U-test. These statistical methods were used in both unmatched and matched groups.

Kaplan–Meier curves were used to estimate survival and freedom from reinterventions in patients undergoing standard contralateral gate cannulation and speed gate cannulation. A bivariate test was used to assess the relationship significance for correlation analysis. Statistical significance was considered to be *p* < 0.05. For Kaplan–Meier, a standard error exceeding 10% was reported. Statistical analysis was performed using SPSS 22.0 (SPSS Inc., Chicago, IL, USA).

## 3. Results

**Figure 4:**
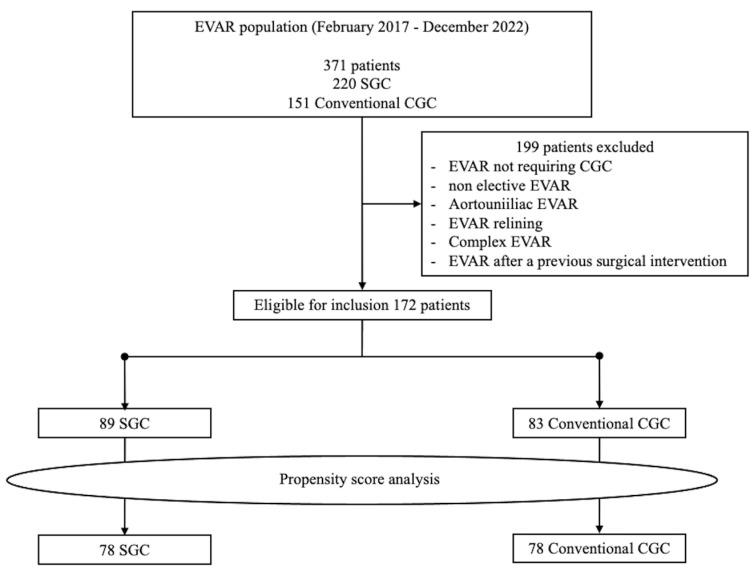
Population flow chart.

After propensity score analysis on 172 patients, 78 patients in the conventional CGC group and SGC group were included for a total of 156 patients (Figure 4).

The baseline characteristics of both matched and unmatched groups are reported in Table 1. Before matching, a significant difference was observed for hypertension, PAD, lipid disorders, preoperative NYHA classification, and aneurysm behavior. After matching, non-significant differences were observed in both standard CGC and SGC groups (Table 1).

In the conventional CGC group, the mean follow-up was 38.77 ± 21 (median: 36; IQR: 24–60) months; in the SGC group the mean follow-up was 37.46 ± 20 (median: 36; IQR: 24–60) months, *p* = 0.17.

After matching, primary outcomes registered no significant differences in overall operative time (73 [67–85] vs. 77 [74–86] min; *p* = 0.07) between the two groups. In the SGC group, a significant reduction was reported for CGC time (4 [1–6] vs. 8 [6–14] min; *p* = 0.001); mean contrast medium (61 [50–72] vs. 77 [71–92] mL; *p* = 0.03); fluoroscopy time (12 [9–16] vs. 17 [12–25] min; *p* = 0.001); DAP (15 [9–21] vs. 26 [16–34] G*cm^2^; *p* = 0.002); and CGC fluoroscopy time (45 [26–65] vs. 96 [70–133] sec; *p* = 0.001) (Table 2).

The aortic neck angulations on coronal and sagittal axes strongly correlate with cannulation time (*p* = 0.02) together with AAA sacciform morphology (*p* = 0.04). A higher cannulation time was associated with a higher neck angulation on coronal and sagittal axes and the presence of a sacciform aneurysm morphology (Table 3).

**Figure 5:**
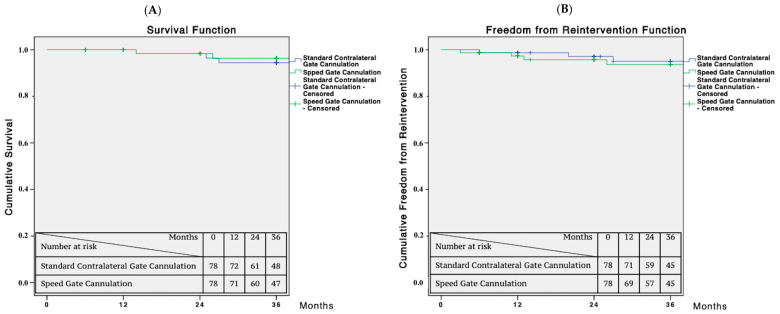
(**A**) Survival and (**B**) freedom from reintervention estimated from 3-year Kaplan–Meier curves for standard contralateral gate cannulation and speed gate cannulation. Standard error does not exceed 10% at 3 years for both survival curves.

The technical success was 100% with no perioperative mortality or type I/III endoleak registered in either group. One perioperative iliac leg occlusion was observed in the SCG group. No differences were observed in the iliac limb components (2 [1,2] vs. 2 [1,2]; *p* = 0.9). During the follow-up, no aneurysm-related mortality or complications were observed. Survival at 36 months was 97% for the SGC group and 96% for the standard cannulation group (*p* = 0.678). Freedom from reintervention at 36 months was 93% in both groups (*p* = 0.834) (Figure 5).

## 4. Discussion

Difficult CGC remains an issue in EVAR procedures to address AAA, even in experienced centers. Difficult CGC has been associated with an increased overall procedure time, CGC time, X-ray exposure, and contrast medium usage [4]. In emergency situations, where time is essential, tools to reduce the time of CGC play an even more important role.

Different anatomic variables have been reported to negatively influence the time required for CGC, including maximal aneurysm diameter, iliac tortuosity, active thrombus-free lumen, and aortic bifurcation angulation [5,6].

In 2019, Pakeliani et al. reported an improved technique for sheath-supported contralateral limb gate cannulation; the only anatomic variable found to correlate significantly with CGC time was the angulation of the aortic bifurcation in the coronal axis [2]. In the present experiment, the anatomic variables correlating significantly with the SGC time were the aortic neck angulations on coronal and sagittal axes and the aneurysm saccular morphology.

Different experiments have reported a correlation between aortic neck angulation and difficult CGC, despite device improvements, and the CGC is still dependent on surgeons’ skills and technical choices [7,8,9,10].

Dang W et al., for a population of 100 consecutive patients treated with EVAR, highlighted the role of angulations on the speed of CGC using a standard cannulation gate technique. They predicted the opening and final position of the contralateral gate to be always near the proximal neck axis [11]. SGS using a supported contralateral sheath aims to provide a closer position to the contralateral gate orifice, to facilitate cannulation. The use of a supporting introducer sheath with an inflatable valve allows simultaneous placement of the coaxial guidewire with no bleeding. In addition, after CGC with SGC, the guide employed to catheterize the CGC is easily exchanged with the supporting stiff wire.

In patients with saccular AAA morphology, we found that the orientation of the contralateral gate was mainly in the direction of the aneurysm sac space, to prevent the gate opening over the aortic wall. Thus, the gate opening orientation did not depend on the best orientation for cannulation with both standard and speed gate cannulation. We argue that this feature was the explanation for the significant correlation between the AAA saccular morphology and higher CGC time.

Also in this series, the CGC time was not influenced by maximum aneurysm diameter and iliac tortuosity. It can be argued that the supported sheath is maintained in the direction of the aortic neck, reducing the possibility of navigating wires and catheters into the aneurysm sac. Moreover, eventual iliac tortuosity is irrelevant due to the introduction of the supported sheath at the level of the aorta.

Additional techniques to facilitate contralateral gate cannulation are available. The crossed-limb technique was reported by Yagihashi K et al. for patients presenting tortuous iliac accesses, to facilitate CGC. However, the crossed limb or “ballerina” configuration does not decrease the time of cannulation [12,13].

The snare technique from contralateral access or the brachial access and consequent trough and trough technique are valid alternatives to achieve technical success but represent a secondary choice due to the more invasive nature and higher risk of dislocation. In addition, higher costs are reported in patients requiring additional maneuvers for anterograde cannulation and subsequent guidewire snaring [4,14,15]

During the follow-up, the SCG was safe with similar results when compared to standard retrograde cannulation in terms of mortality and patency. The SGC allowed a reduction in CGC time, mean contrast medium, fluoroscopy time, radiation exposure, and CGC fluoroscopy time. The SGC was not associated with an increased overall procedural time when compared to standard retrograde gate cannulation.

Overall, the SGC technique facilitates cannulation with the most popular bi/tri-modular stent-grafts available; in our experiments, the SGC allowed us to address several complex AAA anatomies, especially in emergency scenarios.

The first limitation of the present study is related to the retrospective nature and lack of randomized control. Despite the matching propensity process allowing two almost identical groups of treatment in terms of comorbidities and anatomic variables, slight differences between the groups persisted. The limited sample size represents the other limitation of the study’s consistency.

## 5. Conclusions

In this preliminary experiment, the use of SGC during EVAR was feasible with no significant registered postoperative complications. The SGC was not associated with an increased overall procedure time but with a significant reduction in contrast medium usage, radiation exposure, and CGC time. Deploying the stent-graft main body in a crossed limb configuration when guidewires are crossed at the level of aortic bifurcation is recommended. The SGC is a simple adjunctive technique, and its use should be considered in standard EVAR, especially in emergency scenarios, where time is of the essence.

## Figures and Tables

**Table 1:** Baseline patient characteristics of unmatched and matched cohort.

	Unmatched Cohort			Matched Cohort			
Variables	SGC (89)	STD (83)	*p*	SGC (78)	STD (78)	SE Diff	*p*
Age, y, (SD)	78 [±8]	81 [±9]	0.16	78 [±8]	81 [±9]	−6.4	0.21
Male gender, n (%)	63 (70.8)	67 (80.7)	0.94	62 (79.5)	63 (80.8)	0.12	1
Associated comorbidities							
Hypertension, n (%)	81 (91)	67 (80.7)	<0.001	65 (83.3)	59 (75.6)	0.07	0.66
Ever smoker, n (%)	48 (53.9)	49 (59)	0.9	47 (60.2)	46 (59)	0.11	0.9
COPD, n (%)	11 (12.3)	15 (18.1)	0.6	10 (12.8)	10 (12.8)	0.12	0.77
CVD, n (%)	8 (9)	7 (8.4)	0.4	6 (7.7)	6 (7.7)	0.14	0.75
PAD, n (%)	11 (12.3)	17 (20.5)	<0.001	10 (12.8)	11 (14.1)	0.12	0.74
Diabetes, n (%)	41 (46.1)	48 (57.8)	0.2	35 (44.9)	27 (34.6)	0.13	0.55
Lipid Disorder, n (%)	63 (70.8)	55 (66.3)	<0.001	57 (73.1)	54 (69.2)	0.15	0.42
CAD, n (%)	44 (49.4)	42 (50.6)	0.3	35 (44.9)	41 (52.6)	0.11	0.58
Previous CI, n (%)	21 (23.6)	27 (32.5)	0.5	17 (21.8)	18 (23.1)	0.05	0.66
Preop GFR < 60 mL/min, n (%)	10 (11.2)	12 (14.4)	0.4	9 (11.5)	9 (11.5)	0.11	0.71
LVEF < 50%, n (%)	13 (14.6)	15 (18.1)	0.4	12 (15.4)	13 (16.7)	0.15	0.75
Preoperative NYHA classification							
I, n (%)	69 (77.5)	57 (68.7)	0.5	60 (76.9)	56 (71.8)	0.09	0.89
II, n (%)	12 (13.5)	16 (19.3)	0.6	11 (14.1)	14 (18)	0.11	0.71
III, n (%)	5 (5.6)	6 (7.2)	<0.001	4 (5.1)	5 (6.4)	0.13	0.77
IV, n (%)	3 (3.4)	4 (4.8)	0.3	3 (3.8)	3 (3.8)	0.08	0.91
ASA classification							
I, n (%)	0	0	1	0	0	-	1
II, n (%)	0	0	1	0	0	-	1
III, n (%)	63 (70.8)	61 (73.5)	0.4	59 (75.7)	58 (74.4)	0.13	0.66
IV, n (%)	26 (29.2)	22 (26.5)	0.5	19 (24.3)	20 (25.6)	0.11	0.74
Aneurysm behavior							
MATD, mm [IQR]	64 [55–71]	66 [55–77]	0.42	65 [55–69]	65 [55–70]	3.7	0.35
Neck length, mm [IQR]	13 [10–19]	15 [11–22]	0.12	14 [10–19]	15 [11–21]	6.8	0.18
Neck angle, ° [IQR]	43 [25–55]	34 [22–41]	0.05	38 [23–40]	39 [24–40]	4.8	0.37
Neck diameter, mm [IQR]	26 [22–29]	24 [20–27]	0.6	24 [21–28]	24 [20–28]	7.2	0.36
Right CIA involvement, n (%)	17 (19.1)	13 (15.7)	0.73	15 (19.2)	12 (15.4)	0.11	0.42
Left CIA involvement, n (%)	7 (7.9)	9 (10.8)	0.65	6 (7.7)	8 (10.2)	0.12	0.39
Fusiform Shape, n (%)	63 (70.8)	59 (71.1)	0.8	58 (74.4)	55 (70.5)	0.3	0.71

SGC: speed gate cannulation; STD: standard cannulation; SE Diff: standard error difference; y: years; IQR: interquartile range; n: numbers; COPD: chronic obstructive pulmonary disease; CVD: cerebrovascular disease; PAD: peripheral arterial disease; CAD: coronary artery disease; CI: cardiac interventions; Preop: preoperative; GFR: glomerular filtration rate; LVEF: left ventricular ejection fraction; NYHA classification: New York Heart Association classification; ASA: American Society of Anesthesiologists; MATD: maximal aneurysm transverse diameter; CIA: common iliac artery.

**Table 2:** Operative details of the unmatched and matched cohort.

	Unmatched Cohort			Matched Cohort			
Variables	SGC (89)	STD (83)	*p*	SGC (78)	STD (78)	SE Diff	*p*
Stents graft fabric							
Endurant, n (%)	64 (71.9)	63 (75.9)	0.42	58 (74.4)	59 (75.6)	0.12	1
Endologix, n (%)	16 (18)	13 (15.7)	0.5	13 (16.7)	12 (15.4)	0.12	1
Zenith, n (%)	5 (5.6)	4 (4.8)	0.3	4 (5.1)	4 (5.1)	0.15	1
Excluder, n (%)	4 (4.5)	3 (3.6)	0.3	3 (3.8)	3 (3.8)	0.13	1
Number of components, n [IQR]	2 [1–3]	3 [1–4]	0.16	2 [1–3]	3 [1–3]	1	0.8
“Ballerina” configuration, n (%)	48 (54)	42 (50.6)	0.54	41 (52.6)	41 (52.6)	0.21	0.71
Operative time, min [IQR]	66 [60–75]	79 [68–85]	0.73	73 [67–85]	77 [74–86]	2.1	0.07
CGC time, min [IQR]	3 [1–5]	12 [7–17]	<0.001	4 [1–6]	8 [6–14]	1.2	0.001
Mean contrast medium, mL [IQR]	55 [45–72]	81 [77–93]	<0.001	61 [50–72]	77 [71–92]	1.3	0.03
Fluoroscopy time, min [IQR]	11 [8–14]	21 [13–23]	<0.001	12 [9–16]	17 [12–25]	1.4	0.001
DAP, G*cm^2^ [IQR]	14 [9–18]	32 [28–36]	<0.001	15 [9–21]	26 [16–34]	0.1	<0.001
CGC Fluoroscopy time, sec [IQR]	45 [27]	96 [32]	<0.001	45 [26–65]	96 [70–133]	1	0.001

SGC: speed gate cannulation; STD: standard cannulation; SE Diff: standard error difference; DAP: dose area product; CGC contralateral gate cannulation.

**Table 3:** Pearson correlation.

		CGC Time			
Aneurysm Behavior			Stents Graft Fabric		
MATD	Correlation	−0.108	Endurant	Correlation	−0.043
	Sig (2-t)	0.48		Sig (2-t)	0.777
Neck length	Correlation	−0.139	Endologix	Correlation	0.164
	Sig (2-t)	0.161		Sig (2-t)	0.281
Neck angle	Correlation	−0.347	Zenith	Correlation	−0.1
	Sig (2-t)	0.02		Sig (2-t)	0.513
Neck diameter	Correlation	0.05	Excluder	Correlation	−0.144
	Sig (2-t)	0.744		Sig (2-t)	−0.344
Right CIA involvement	Correlation	0.1	Number of components	Correlation	−0.22
	Sig (2-t)	0.513		Sig (2-t)	0.146
Left CIA involvement	Correlation	0.154	“Ballerina” configuration	Correlation	−0.093
	Sig (2-t)	0.312		Sig (2-t)	0.544
Sacciform Shape	Correlation	0.387	Operative time	Correlation	−0.071
	Sig (2-t)	0.04		Sig (2-t)	0.644

## Data Availability

The data presented in this study are available on request from the corresponding author.
